# Depicting adverse events in cardiac theatre: the preliminary conception of the RECORD model

**DOI:** 10.1186/1749-8090-8-51

**Published:** 2013-03-19

**Authors:** Haralabos Parissis, Lorraine Mc Grath-Soo, Bassel Al-Alao, Alan Soo

**Affiliations:** 1 Cardiothoracic Department, Royal Victoria Hospital, Grosvenor Rd, Belfast BT12 6BA, UK; 2 Beaumont Hospital, Beaumont Road, Beaumont, Dublin, Ireland

**Keywords:** Human factors, Cardiac surgery

## Abstract

Human error is a byproduct of the human activity and may results in random unintended events; they may have major consequences when it comes to delivery of medicine. Furthermore the causes of error in surgical practice are multifaceted and complex. This article aims to raise awareness for safety measures in the cardiac surgical room and briefly “touch upon” the human factors that could lead to adverse outcomes. Finally, we describe a model that would enable us to depict and study adverse events in the operating theatre.

## Introduction

Human factors (HF) is concerned with the ‘fit’ between the user, equipment and their environments. It takes account of the user's capabilities and limitations in seeking to ensure that tasks, functions, information and the environment suit each user. With other words HF is a concept that encompasses the science of understanding the properties of human capability, the application of this capacity to the design and engineering and lastly the capability of ensuring optimal application of human factors integration to a program.

Analysis of HF is a discipline that spans various fields and emerges specifically in response to the safety concerns of multi high-risk industries. Although theoretically based, it has a resolutely practical emphasis, always aiming to bridge the gap between theory and application. HF thinking is now applied to healthcare in a variety of ways. Recently, a report by The World Health Organisation [1] published a 19-item Surgical-Safety-Checklist, which outlines safety checks and recommends performing at specific-task of the patients surgical journey.

The aim of checklist-implementation was to change surgical-systems and team -behavior.

### Behavioral outcomes in the operating theatre

Optimal outcome following an operation is a result of multimodal harmony of variables. Attitude, team work, organization and protocol implementation as well as optimal knowledge of the task referred to, are important as simply seen when one defines the difference between mistake an error: A mistake which means “wrongly taken” is a wrong response that if you thought about it you would realize is wrong. An error which means “to wander or stray” is a wrong response because you have no knowledge about what the right answer is. With other wards a person if given a second chance has the potential to correct a mistake, whereas a person has no potential to correct an error until he learns what is correct.

In such a volatile environment mistakes and their precursors are waiting to occur; More than 50% of surgical adverse events occur in theatre [2] whereas half could have been prevented with simple-measures. As per Wiegmann [3] about 60% of surgical errors were picked up and managed immediately by the surgical team. It is interesting that of these errors, 32% were events during which the team experienced difficulty performing a specific technique or procedure.

Adverse events are caused by decisions taken by persons at the delivery end of a system or are results of deficiencies in the organizational and management level of the system.

Surgical error may occur when lack of skill or ability is combined with behavioral deficiency. What we mean with this statement? Well, when the knowledge or the skills required for a given procedure are lacking and despite this, for example the leading surgeon is deciding to undertake the procedure with no safety backup. With other wards lack of appreciation of ones knowledge limitations can lead to misjudgment and adversity.

Skill-based errors were thought to be the most common type of unsafe acts in theatre [4] followed by changing or bending the predetermined rules in under pressure circumstances.

Reasons “swiss cheese” model illustrates that the organizational influence (climate, resource management, and policies) impact supervisory processes (scheduling, training, and oversight), which in turn establish the preconditions (technological and teamwork related) that produce errors.

The Human Factor Analysis Classification System (HFACS) framework was developed using over 300 naval aviation accidents obtained from the US Naval Safety Center.

Based on HFACS, questions were developed to target each of the causal categories within the HFACS by ElBardissi et al. [4]. With other words this group have tested the application of HFACS in Cardiac surgery. When asked in an open-ended manner to characterize surgeon personality and its reflection in the operating theatre environment, 35% of individuals stated that surgeons are arrogant or demeaning, negatively affecting team function, 24% stated that surgeons have a strong or intense personality, also negatively affecting team function. Therefore, in this report with regards to teamwork issues, 59% of responders indicated that a surgeon’s personality (by means of surgeons misbehavior in theatre), negatively affects the way the surgical team functions, Because the surgeon’s leadership and team-ness are hugely important [5], many institutions have attempted to address this issue of occurred behavior by sending surgeons to charm school in order to improve their attitude in the operative room; this is an interesting point and has to be taken seriously from our senior colleagues. However, the exact role of teamwork in error causation is unclear, emphasizing the importance of additional research in this area before implementing interventions that have no scientific basis.

Although it is unlikely that one’s innate personality can be changed it is undoubtedly possible to alter aspects of behavior, which impact negatively on colleagues and on the team in the work place. With appropriate training, individuals can improve and thus function more effectively as part of a multidisciplinary team.

Major and minor events represent various types of human errors. Those events have been elegantly defined and extensively studied [6]. Minor events are errors that alone were not expected to have serious consequences for the patient; these included instrument handing, errors by the scrub nurse, or communication problems in the theater team. Major events are more serious errors, such as for example, accidental injury of a coronary artery and severe laceration of the aorta during canulation.

The surgeon’s diagnostic skill, knowledge of the various maneuvers to rectify problems, and communication with the team are important prerequisites of compensation. Uncompensated major events have a detriment outcome. If each of them is appropriately compensated, however, death can be avoided. Minor events are different. They are less apparent and insidious, and many of them are not even noticed by the operators. They are independent of case complexity and correlate with “lack of flow”, distractions and lack of communication in the operative theatre. More specifically Barach et al. [6] in a prospective observational study of one hundred two patients undergoing pediatric cardiac surgery, they analyzed minor and major events; the surgeon wore a video head-camera so that observers could also follow the surgical steps. The observers created a handwritten observational tool to document the surgical flow. A median of 1.1 major events (range 0–6) occurred per case; with 43% of the major events observed to occur when the aortic cross clamp is release and is followed by weaning and separation from CPB. A median of 18.3 minor events (range 2–54) occurred per case, most often during CPB. This study revealed that adverse events occurred regularly during cardiac surgery and moreover that case complexity and duration were significant predictors of major adverse events, which in turn correlates with adverse outcome. On the other hand, reactive than pre-emptive compensatory mechanisms were detected in 98% of major events and 90% of minor events.

### The RECORD model

In order to identify the failed or absent defenses on the chain that leads to unsafe acts and sentinel events we hypothesize and presenting here a model that maybe have a practical application in safe delivery of surgical care.

We need to outline here that this is a preliminary report of the model, which recently started to evaluate its feasibility in practice.

The model proposed is based in the abundance literature that suggests that surgical practice should be reported, evaluated and one should reflect on it.

Furthermore the model takes into consideration the visual grading of a procedure; either with the use of “human observers” specialists to the procedure in question able to grade it, or the use of a video camera recording.

Lastly the model aim to use two more tools: audit forums and morbidity and mortality meetings and the use of questioners with a five point Likert scale based on frequency of occurrence (always= 5, very often= 4, sometimes= 3, rarely= 2, never= 1).

We are currently enrolling 100 CABG cases in an attempt to answer two aspects:

Firstly, we will be looking into reporting unsafe acts (Classified as per HFACS) by means of registering various errors:

1) “Thinking” errors as intended behavior that proceeds as designed, yet the plan proves inadequate for the situation.

2) Poorly executed procedures,

3) Improper choices

4) Misinterpretation of relevant information.

5) Skill-based errors: by examining the technique with which one performs a task. These errors frequently appear as forgotten intentions, and omitted items during procedures.

6) Perceptual errors: Errors arise when sensory input is degraded. Faced with acting on imperfect or incomplete information, OR staff run the risk of misjudging procedures as well as responding incorrectly to a variety of stimuli.

7) “Bending the rules.” This type of violation is habitual by nature.

And secondly we will be evaluating if CABG surgery in practice is adherent to principles and local protocols. The next step would be to add a questioner to target each of the causal categories within the HFACS structure:

1. Organizational structure and Resource management.

2. Structural supervision: management of personnel, and resources, including training, guidance, and leadership.

3. Problem correction: Instances when deficiencies among individuals, equipment, training, or other safety areas are “known” to the supervisor, yet allowed to continue.

4. Inappropriate operations: Management of work, including aspects of risk management and crew pairing.

5. Technological environment: Design of equipment and controls, display-interface characteristics, checklist layouts, task factors, and automation.

6. Physical environment: The operational setting and the ambient environment, such as heat and lighting.

7. Adverse mental states: Psychological and (or) mental conditions, such as fatigue, pernicious attitudes, and misplaced motivation, that negatively affect performance.

8. Adverse physiological states: Medical and/or physiological conditions such as illness, intoxication, and pharmacological or medical abnormalities known to affect performance.

9. Physical/mental limitations: Physical/mental disabilities, such as poor vision, lack of skill, aptitude, knowledge, and other mental illnesses.

10. Teamwork: Communication, coordination, and other teamwork issues

11. Personal readiness: Off-duty activities and other mandates, required to perform optimally.

The last step of the model would be to use a Video camera recording randomly and human factor specialists.

**Figure 1:**
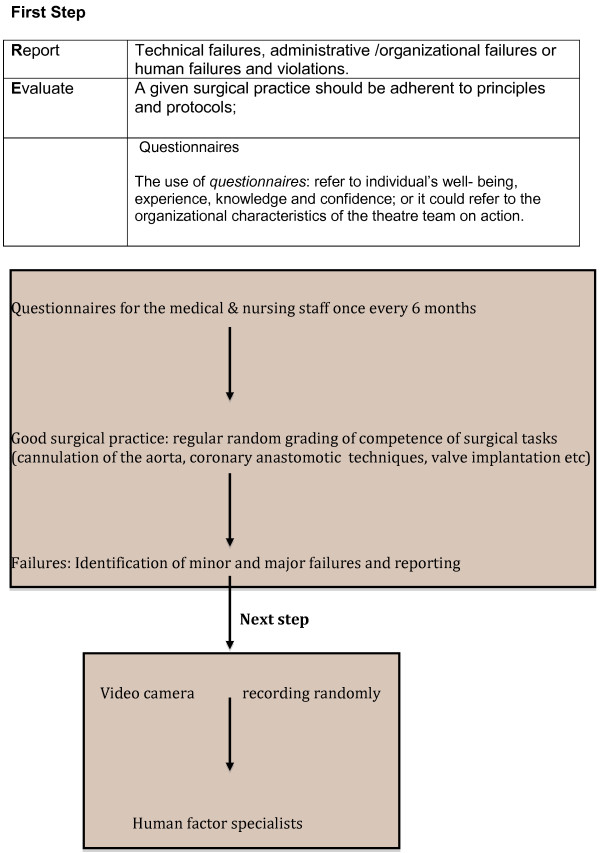
An algorithm for investigating adverse events in the operating theatre.

Implementation all the steps of the RECORD model may be cumbersome and preliminary results are not out yet however we believe that this multifaceted approach may allow us to capture and study “ adverse events in cardiac theatre” in order to improve HF. In Figure 1 an algorithm to stepwise investigations of adverse effects in theatre is given. In Table 1, the principles of the RECORD model are depicted.

**Table 1:** Depicting and studying “events in theatre” in order to improve human factors: the “RECORD” model

**The “RECORD” model**	
**R**eport	In Incident **R***eporting* systems, error should be categorized according to the source of it; so errors can be occur due to technical failures administrative and organizational failures or human failures and violations. However regardless of its source, the correct approach to human error recognizes the need for reporting and reflecting on them. Analysis of human factors means encouraging error reporting in a non-punitive environment, where it is seen as a valuable source of information, facilitating education and future error prevention.
**E**valuate	Constant grading of theatre activity can be achieved by **E***valuation* as to whether a given surgical practice is adherent to principles and protocols; for example prior to chest closure the team asks the anesthetist to make sure that the Swan-Ganz catheter is mobile and is not “caught up” with the atrial sutures; or when requesting for a IABP support the balloon size has to be double checked by perfusionists and surgeons; A patient with an air leak who comes to theatre for a VATS pleurectomy should not have the drain removed up till the chest is open for the simple risk of tension pneumothorax.
**C**entralize	Creation of a “**C***entralized* cardiac registry” of major events and near misses, whereby incidences in cardiac theatres should be reported and should be used as “learning examples for avoidance”.
**O**bserve	**O***bservers*: “Human factor specialists” should be experts to the procedures involved: this is an important concept because a commonly predictable event such as for example, inability to be weaned off Cardiopulmonary bypass first time due to air embolism in the coronaries could be criticized as a near miss event from an inexperienced eye.
**R**ecord with Video camera	Video camera **R***ecordings*; it is been used in other specialized areas such as during performance of highly skilled actions in aviation. It is somehow difficult to appreciate as to how it could be an established monitoring tool in the operating theatre, however one has to appreciate that it provides an objective index of a performance and a reflecting tool to refer to.
**D**ual action: Audit and Questionnaires	Regular clinical *audit forums* whereby the current surgical practice is scrutinized against protocols; this is an essential component of the “smooth implementation” of surgical practices and provides room for improvement by identifying human errors and eventually correcting them by “closing the audit loop”.
	The use of *questionnaires*: They are helpful in depicting surgical practices. A direct questioner to the surgical team could potentially refer to individual’s well- being, experience, knowledge and confidence; or it could refer to the organizational characteristics of the theatre team on action. Human factors questionnaires should be “filled in” before or during the procedure, but not afterward, to prevent hindsight bias.

“RECORD” stands for:

**R: R**eport, **E: E**valuate, **C**: **C**entralize, **O: O**bserve, **R**: **R**ecord with video.

**& D**: **D**ual action: Audit and questionnaires’.

## Discussion

In an elegant study by de Leval et al. [7], a table of hypothetical perioperative scenarios with a various types of errors in the cardiothoracic operating environment are presented, in a didactic way. The authors, outline the fact that error detection should be the first step in error handling; moreover, that there is a suggestive evidence on the direct influence of the number of minor events per case and their impact to adverse outcomes.

The Northern New England Cardiovascular Disease Study Group is a learning collaborative, that has completely devoted to improving performance of cardiac surgery. By using the tools that we have alluded to, albeit focusing on quantitative outcome data they have been able to produce the best outcomes across the entire 12 hospital network (Full publication list at: http://www.nnecdsg.org/pub_lit_2.htm).

Furthermore there are a number of publications whereby various authorities have done an extraordinary amount of work on improving adult and pediatric cardiac surgery performance and should be cited here [8,9]. The authors summarize the lessons that have been learned about critical incident and near-miss reporting in other high technology industries that are pertinent to cardiac surgery.

Critical incident and near miss reporting that is based on human error taxonomies is in its infancy in the field of cardiac surgery [8]. However, monitoring near misses [7] can provide early indication of deterioration in surgical performance. Furthermore it is important to outline that the hospital with the highest mortality rate [8] also had a high failure-to-rescue rate, suggesting that there were problems in the management of difficult complications.

Failure to rescue a patient might therefore be an appropriate measure of “inadequate organizational performance”. A “Centralized cardiac registry” of major events and near misses, whereby incidences in cardiac theatres should be reported and should be used as “learning examples for avoidance” may become a helpful tool.

In a series of 24 successful operations Catchpole et al. [10] 366 failures when check lists, notes and video recordings were employed. Interestingly, skill, knowledge and decision making failures were only a small percentage of the failures; furthermore Longer and more risky operations were likely to generate a greater number of minor failures than shorter and lower risk operations

The same authors [11] by using a validated scale adapted from research in aviation they looked at the ability of a team to work safely; they concluded that decreasing the number of minor problems can lead to a smoother, safer and sorter operation.

Schraagen et al. [12] in an elegant report and rigorous work they trained human factors observers to observe and code the non-routine events and teamwork from time of arrival of the patient into the operating room to the patient handover in the intensive care unit. The authors concluded [13] that their suggested trained model it is ideal to explore team performance.

Using trained human factor observers [14] 40 paediatric cardiac cases were observed using both quantitative and qualitative measures. The important results of this study showed that surgeons displayed better teamwork during complicated procedures and also that more procedural non-routine events were associated with more complicated postoperative course.

Bognar et al. [15] using the power of a survey amongst paediatric cardiac surgery team members found that Staffing levels, equipment availability, production pressures, and hectic schedules were concerns. More interesting responders confessed that guidelines and policies were often disregarded.

It has wrongly been perceived by few [16], that surgical skills are innate aspects of ones personality; they can neither be taught nor acquired. In this context, we should differentiate between ‘innate ability or aptitude’, which an individual is born with and brings to particular tasks, and ‘skill’, in execution, which is acquired by training and reinforcement. Furthermore, whilst some individuals seem to be able to acquire these skills easier, many others could have these skills improved by lengthier training. On their own, however, knowledge or skills are not enough. For example, there are trainee surgeons who are well informed on the surgical literature but are less than adequate in the operating room. Contrary, there are surgeons whom are technically expert but consistently fail to get the results that would be expected from such expertise.

Surgeons with the “right knowledge and technical expertise” get better outcomes because they operate on the right patients the right time, continue to perform under stress and they manage to successfully harness the support of a multidisciplinary team to get the best results. It is well known that Crew resource management skills (how to manage/guide the theatre team) are particularly important for cardiac surgeons. This is one of the WHO checklist emphasis points.

It has been hypothesized [17] that only 25% of the important events, which occur during a surgical procedure, are related to manual-technical skills and that 75% relate to decision-making (especially during crises), communication, teamwork and leadership. Other HF, which are important in surgical practice, include self-awareness (i.e. insight), conflict resolution and error management. For example ‘task conflicts’ the “who, what, when and why” between the various theatre teams should be settling easily without escalation to an argument.

Causes of accidents in aviation industry are primarily related to deficiencies in nontechnical skills, rather than a lack of technical expertise. Flin et al. [18] in an elegant review elaborates on Crew Resource Management (CRM) courses as an educational tool to improve aviational safety; The four primary categories subdivide into two social skills (Co-operation; Leadership and Management) and two cognitive skills (Situation Awareness; Decision Making). Lack of nontechnical skills, have also been studied during surgical procedures, with great interest [13].

In this editorial we are projected an idea based in numerous and very important research in the topic of Human Factors. We are proposing a local and also a national registry of “events”. That could be subdivided as per “severity of the events”. In addition, we are taking into consideration simple reporting mechanisms-questioners, together with real perioperative data acquisition. That may enable us to scrutinize surgical practice and also test the implementation and validity of cardiac surgical protocols.

### Limitations of applying “various systems” in clinical practice

Under-reporting [19] of incidents is a limiting factor that unfortunately it relates to the old notion of “a blame culture” in medicine; it is inappropriate, when junior doctors do not report incidents [20] because they fear they will be blamed. Equally it is wrong when they are blamed by senior colleagues; under the circumstances the team should be united to investigate and learn from the errors.

The obligation to name and define the role of the person reporting the incident can be a restrictive factor for reporting; it is apparent that confidentiality encourages an easier reporting procedure and probably should be implemented when possible. Moreover, the retrospective nature of reporting incidences further necessitates their need for validation and accuracy.

Clear definitions of [8] near misses in cardiac surgery is complicated by the need to distinguish between those events against serious peri-operative and post- operative complications. This can only be achieved when the human factors observers are trained and familiar with the procedures under investigation. With other words standardized training and calibration of observers would improve the data collection.

The capture of adverse actions and events are subjective and depend on the observers training and education; therefore, depicting accurately data in this respect could be limiting by interpreter reliability. Moreover, there are many technical challenges when video taping surgical teams such as logistics, ethics and interpretation [12]. Weaknesses in using video for data include lengthy video review processes, poor audio, and the inability to adequately analyze events outside the field of view [21].

The development and implementation of a system for measuring technical performance in the operating theatre is difficult. The challenges of grading the technical expertise, has been addressed by Karamichalis et al. [22]. An individual practitioner’s surgical performance includes the technical domain and other nontechnical skills such as cognitive flexibility, decision-making etc.

Technical performance, is defined as “the adequacy of the surgical anatomic repair intended,” and as per Larrazabal et al. [23] intraoperative technical performance is one of the most important, if not the most important, parts of the therapeutic process and determines postoperative outcomes. None of the currently available quality monitoring tools measures technical performance; the authors created and validated a scoring tool in this respect; the limitations of their tool is however due to the fact that in their model “rating surgical adequacy” is based on ECHO, which creates the biases of the operator dependent technique.

Lastly, using models for reducing errors may be part of the solution; as per Auroy et al. [24] risk assessment and control require analysis of both outcomes and process of care.

We admit into several limitations of this report. The model reported here is more of a zealous and enthusiastic project, in an attempt to capture more accurately, the already well defined many aspects of human factors in the cardiothoracic environment.

The first attempt of implementing a robust model was conceived by our team, following few years of using Briefing and debriefing in the cardiac operating room, in a manner similar to the model reported by Papaspyros et al. [25] with satisfactory outcomes (unpublished results).

We need to clarify here, that we conceived the idea of the RECORD model in an attempt to put more variables together in a continuous fashion. With other words, we would try to compile a “feedback approach” by the staff involved in the theatre environment together with the “specialists reporting approach” of the human factor observers in a stepwise fashion.

We would also like to confess, that we are still working to make the questioners simple and reproducible, for easy use.

So overall, this is a preliminary idea that we are working to materialize.

Lastly, in this editorial we hypothesized that putting more variables together we will be able to overcome some of the limitations to human factor research and analysis of performance assessment; although we just initiate implementing our model in clinical practice we hope that we will be back with real world data analysis in order to be able to depict and act upon clusters of latent errors before they become active. We are hoping to show in a subsequent study by piloting this model, that we will be able to support the basic idea of the model (more variables, more feedback, less bias) for others to emulate.

## Conclusions

The ability to manage errors and unexpected events successfully during an operation is a marker of surgical excellence.

By enlarge, imminent errors include such factors as fatigue, communication or patient related factors, such as a difficult intubation, while systemic errors concern organizational matters such as shift patterns and staffing.

If we are pursuing perfection, then we ought to review and reflect in each case, both early after the operation and later in follow-up, to determine which aspects of the management could be improved. The RECORD model is a “conceived idea” that is taking into consideration all the hard work that has been done in the field of human factors the last 25–30 years. We are hoping to finalize and validate the model under the principles of: use simple variables, straight forward feedback real time observational approach with elimination of bias; however the model needs to be clinically implemented, before we conclude that may improve clinical governance.

## Competing interest

The authors declare that they have no competing interests.

## Authors’ contributions

HP conceived the idea and drafted the manuscript, in all the various phases and modifications of it. LMGS carried out an extensive literature review. BAA participated in the sequence and drafting of the manuscript. BAA also had some valuable ideas in the construction of the RECORD model. AS participated in the overall design and coordination of the study; also helped to draft the “discussion” and “limitations” on the manuscript. All authors read and approved the final manuscript.
